# Seizure manifesting as a reaching/grasping movement in a patient with post‐traumatic epilepsy

**DOI:** 10.1002/ccr3.1872

**Published:** 2018-10-17

**Authors:** Toru Horinouchi, Kotaro Sakurai, Tsugiko Kurita, Youji Takeda, Yusuke Yoshida, Hisashi Akiyama, Katsuyuki Fukushima, Ichiro Kusumi

**Affiliations:** ^1^ Department of Psychiatry and Neurology Hokkaido University Graduate School of Medicine Sapporo Japan; ^2^ Department of Psychiatry Hokkaido Prefectural Koyogaoka Hospital Abashiri Japan; ^3^ Department of Psychiatry National Hospital Organization, Obihiro Hospital Obihiro Japan; ^4^ Fukushima Neuro Clinic Nanae Japan

**Keywords:** anterior cingulate cortex, long‐term EEG, reaching/grasping movement, seizure video, supplementary motor area

## Abstract

Electrical stimulation (ES) of the pre‐supplementary or cingulate motor area can cause reaching/grasping (R/G) movements with the hand contralateral to the side of the brain receiving the ES. We report this phenomenon occurring in a 23‐year‐old right‐handed man during spontaneous epileptic seizure, which developed after traumatic brain injury.

## 1 INTRODUCTION

Epileptic seizures may present symptoms of ictal grasping. Since there are various types of ictal grasping, efforts have been made to classify them according to their respective characteristics.^1^ Frontal hyperkinetic seizure has been reported as a repetitive and compulsive movement, in which the patients grabs his/her body or clothing alternately with both hands; it is often observed in patients with frontal lobe epilepsy.^1^ Moreover, frontal ictal symptoms that lack repetitive or compulsive characteristics, resembling reaching movements with subsequent whole hand prehension, in the air, has been reported.^2^ Furthermore, the duration of ictal grasping in patients with temporal lobe epilepsy has been reported to be relatively long and to be accompanied by oro‐alimentary automatisms.^1^ An accurate understanding of ictal grasping as a seizure symptom is thus important for inferring epileptogenic zones from symptomatology.

Although electrical stimulation (ES) can elicit motor symptoms, the following elicited symptoms have not been reported in association with spontaneous epileptic seizures to date. ES of the pre‐supplementary motor area (pre‐SMA) or the cingulate motor area (CMA) has been reported to result in a specific type of movement called a reaching/grasping movement (R/G movement). It involves the irresistible urge to grasp something in the visual field, followed by the act of grasping it with the hand contralateral to the side of the brain that received the ES.^3^ In a previous report, 1 out of 9 ESs of the anterior CMA ventral bank (CMA_av_), 2 out of 13 ESs of the anterior CMA dorsal bank (CMA_ad_), and 1 out of 34 ESs of the pre‐SMA resulted in R/G movements.^3^

Here, we report the case of a patient with epilepsy who presented an R/G movement, which we considered to be part of a spontaneous epileptic seizure. The R/G movement was the only motor expression of every single seizure. Additionally, unlike in the previous studies, the R/G movement was clearly guided by visual sensation and was not an automatic movement to grasp an object nearby without visual input. To our knowledge, R/G movements have not been reported as part of an epileptic seizure to date.

## 2 CASE REPORT

The patient, a 23‐year‐old right‐handed man, showed normal neuropsychological development. He had worked as a jockey and was thrown from a horse in 2007. The accident resulted in multiple traumatic brain injuries, including a left subdural hematoma, traumatic subarachnoid hemorrhage, and a diffuse axonal injury. The patient received therapeutic hypothermia and cerebral decompression, followed by cranioplasty and placement of a ventriculoperitoneal shunt to treat hydrocephalus. However, the patient suffered from sequelae, including right hemiplegia, right homonymous hemianopsia, and severe neuropsychological dysfunction. In 2009, the patient developed epilepsy; the patient experienced right‐sided convulsive seizures and partial seizures that evolved into secondary generalized tonic–clonic seizures (sGTCs), several times. Combination treatment of valproic acid, phenytoin, and carbamazepine was able to suppress the sGTCs, although the patient suffered from other complex partial seizures (CPSs) that resulted in consciousness impairment lasting from approximately 10 seconds to several minutes.

From 2010, the patient repeatedly displayed the symptom of suddenly reaching out with his left hand toward someone nearby and grasping the person's arm or neck. This symptom led him to consult an epileptologist, who treated him with several anti‐epilepsy drugs (AEDs); however, the patient did not respond to this treatment. In 2015, the patient was referred to our hospital and hospitalized for further examination and treatment.

**Figure 1:**
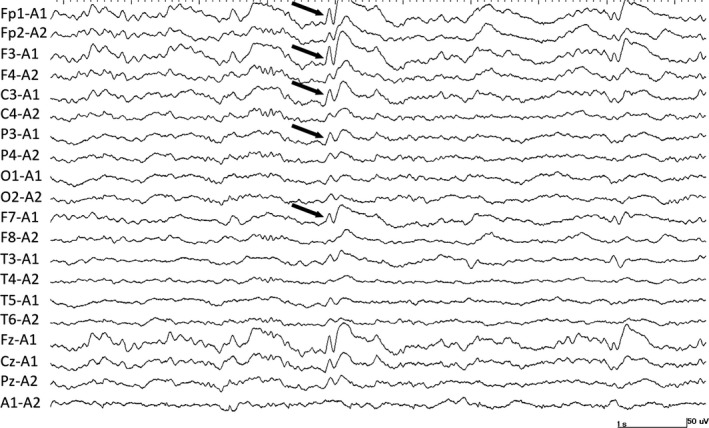
Interictal discharge. Repetitive sharp waves are observed predominantly in the left frontal area during sleep

Neurological examinations revealed right‐sided hemiplegia, which was scored as 1 after manual muscle testing (MMT). Electroencephalography (EEG) revealed repetitive sharp waves, predominantly in the left frontal area, during sleep, which were thought to be interictal discharges (Figure 1). Additionally, pseudocontinuous 3‐Hz slow waves were observed in the left frontal area upon awakening.

We also repeatedly observed the symptomatic behavior in the patient. He reached out with his left hand to persons nearby and grasped and continually twisted the person's hand, neck, or clothes. He sometimes tried to grasp someone by leaning with his body when the target person was located a little far from him. We considered this symptom to be an R/G movement. This behavior tended to be caused by direct somatosensory stimulation, such as a nurse wiping his body, handing him an object, or supporting his body to transfer him from the bed to a wheelchair. The people grasped were visually perceived by the patient and the movement was not an automatic movement, without visual input. His right arm presented a dystonic posture during the R/G movement, despite an MMT score of 1. During this movement, the patient grimaced, which was occasionally accompanied by lacrimation, and consistent repetition of phrases such as “It's enough” and “I'm not sure.” We observed that his eye movement followed the target object movement, and noted that the R/G movement rarely happened when we blocked his vision by placing a towel over his eyes.

The onset and offset of the R/G movement were clear, and the average duration ranged between several seconds and several minutes in the absence of a stimulus of the symptom, such as continued somatosensory stimulation. While the patient could not recall his actions during the R/G movement, he often guessed what had happened and apologized immediately afterward (Video S1, Table 1). We found that his consciousness was altered, because he could not respond at all during the R/G movement. Likewise, he could not recall any word when asked to recall a given word during the R/G movement episode. The patient also exhibited a partial convulsion localized to the right leg for several seconds, which we concluded to be a part of the seizure, despite the right‐sided hemiplegia, although the date of onset of this symptom was not determined.

**Table 1:** Characteristics of this patient’s symptoms

Item	Explanation
Onset/offset	Sudden
Left arm	Reaching/grasping movement
Right arm	Dystonic posture, despite of MMT 1
Expression	Grim, sometimes shed tears
Utterance	Same phrases like “It's enough,” “I'm not sure.”
Inducer	Somatic sensation
Duration	From several seconds to minutes
Frequency	More than 10 times a day

**Figure 2:**
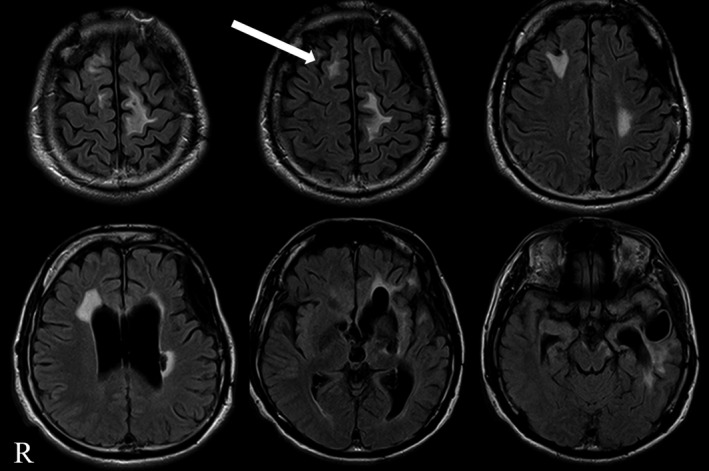
Brain MRI (1.5 Tesla, Fluid‐Attenuated Inversion Recovery; FLAIR). Brain MRI shows multiple old brain contusions, mainly in the left hemisphere. Additionally, several high‐intensity areas are observed, including an area in the right pre‐supplementary motor region, which is indicated by the white arrow

In a long‐term (4‐day) EEG, we were not able to detect any obvious ictal discharge, except for the electromyogram (EMG) during the R/G movement, although we observed the movement more than 10 times during this period. Magnetic resonance imaging (MRI; Figure 2) revealed atrophy and high‐intensity areas in the fluid‐attenuated inversion recovery scan in the left temporal pole, the interior surface of the left frontal lobe, the left basal ganglia, and the bilateral medial frontal lobe. In particular, a high‐intensity area, which appears to be an area of degeneration due to trauma, was observed in the right pre‐SMA, which could have been related to the R/G movement. We finally concluded that the patient's R/G movement was an epileptic seizure caused by traumatic brain injury, and was not a psychiatric symptom. Notably, the sudden onset and offset of the symptom and the pattern and repetition of the symptom strongly support the theory that the symptom was part of an epileptic seizure.

A treatment plan of AEDs, including lamotrigine, levetiracetam, clobazam, topiramate, valproic acid, gabapentin, phenobarbital, perampanel, and lacosamide (in turn) has been maintained for more than 2 years, along with vagus nerve stimulation (VNS). We found that the R/G movement persisted, although the number of R/G movements decreased slightly from more than 30 times a day before treatment to 2‐20 times per day after treatment.

We obtained consent from the patient and his parents for the use of his clinical information, including the seizure video, after we explained that the patient's private information would be completely protected and there would be no disadvantages if they chose to decline this proposal.

## 3 DISCUSSION

The patient, who developed post‐traumatic epilepsy after being thrown from his horse, demonstrated repeated specific movement, considered to be an epilepsy‐associated R/G movement, during which he reached with his left hand toward nearby persons, guided by visual sensation, and grasped them. The seizure persisted despite various treatments.

### 3.1 Comparison to previously reported R/G movements

Reaching/grasping movement as a result of ES has been reported by a single research group.^3^, ^4^ These studies describe the R/G movement as an irresistible urge to grasp something after ES; this leads to exploratory eye movements, scanning the visual field, and reaching out and grasping with the hand contralateral to the side of the ES, under visual guidance. In a previous report, functional MRI revealed that the bilateral CMA and SMA were activated during the act of reaching out and grasping objects.^5^

In our patient, the core symptom of suddenly reaching out and grasping objects under visual guidance corresponded exactly with these previous ES studies and strongly suggests that this symptom involves the same module. The urge to grasp in our patient appeared to be quite strong, as demonstrated in Video S1. However, we could not confirm this directly due to the impairment of consciousness of the patient during CPS. Based on the previous ES study, grasping with the left hand was likely to result from an epileptogenic zone located in the right CMA or pre‐SMA.

Nonetheless, we noted that several aspects of the symptomatic behavior did not correspond to those described in the ES study. The symptom reported in the ES study did not include a dystonic posture, emotional changes, or utterance, and the study did not specify that the object that was grasped was specifically human. However, we detected multiple brain contusions on MRI examination of our patient, including contusions in the left basal ganglia and the left temporal lobe, which are likely to explain the presentation of CPS, dystonic posture, and emotional changes that were observed.

### 3.2 An epileptic seizure

We concluded that the characteristics of the R/G movement indicated that the movement was an epileptic seizure. Most importantly, the patient consistently demonstrated the same manifestation, including a sudden onset and offset, an R/G movement of the left arm guided by visual sensation, a dystonic posture of the right arm, grimacing occasionally accompanied by lacrimation, stereotypical utterances, disturbance of consciousness, and a short duration. Additionally, several certified epileptologists directly observed this symptom and considered it to be an epileptic seizure. We also concluded that the patient did not exhibit psychiatric symptoms. If the R/G movement was a psychiatric symptom, other manifestations, including weaker symptoms, such as displeasure, denial of treatment, or a loud voice, as well as stronger symptoms, such as beating or kicking, could be expected. However, we did not observe any weaker or stronger symptoms during the patient's hospitalization of over 2 years. Additionally, we considered that the patient did not exhibit stereotypy, as stereotypy cannot lead to impaired consciousness, and the patient exhibited consciousness disturbance during each occurrence of the R/G movement.

The R/G movement that the patient demonstrated was partly similar to the alien hand syndrome (AHS), but we considered that they were not the same. AHS includes the failure to recognize ownership of one's limb when visual cues are removed, a feeling that one body part is foreign, personification of the affected body part, and autonomous activity that is perceived as outside of voluntary control or as alien‐induced.^5^ Although the condition is rare, it has been reported that AHS appeared as an epileptic seizure.^6^, ^7^ A patient with AHS usually tries to hinder the AHS movement,^8^ but the patient in the current report never demonstrated any attempt at hindering such movement and even attempted to grasp someone by tilting his body to overcome distance. Thus, his left arm was not only an affected part, but his whole body was affected by the R/G movement.

We were not able to detect ictal discharge during the long‐term EEG. However, if the focus was in the mesial frontal lobe, including the right pre‐SMA, the lack of ictal discharge in the scalp EEG would be consistent with a previous report in which slightly more than 50% of the seizures were obscured or showed no EEG change when the focus was in the mesial frontal lobe of epilepsy patients.^9^ Therefore, we concluded that the lack of ictal discharge in the scalp EEG did not prove that the patient's manifestation was not an epileptic seizure.

### 3.3 Differentiation from other epileptic seizures

Gardella et al classified the ictal grasping symptoms that occur during an epileptic seizure as follows: frontal hyperkinetic seizure is a forced and repetitive movement involving grasping contracture of the patient's body, accompanied by wild bimanual and bipedal movements; frontal lobe seizure was characterized by prolonged grasping of something nearby, several times, without other movements; temporal lobe seizure tended not to be forced and was repeated with a long latency; and extra‐frontal/extra‐temporal seizure differed from the others.^1^ Although the short latency and forced movements observed in this case were similar to the description of a frontal hyperkinetic seizure and a frontal lobe seizure, we judged that the R/G movement that we observed in this case did not belong to any of these classifications for the following reasons.

First, the R/G movement in this case was the only seizure expression, and was not accompanied by any other movements. Secondly, the R/G movement was clearly visually guided, which has not previously been reported as a characteristic of ictal grasping. Thirdly, grasping during the R/G movement was specifically limited to a nearby person's body or clothes. Hence, we believe that the R/G movement in this case was different from the previously reported ictal grasping.

### 3.4 Epileptogenic zone

Based on the presumption that the R/G movement in this case was an epileptic seizure, the epileptogenic zone should be located in the right hemisphere, that is, contralateral to the left R/G movement. However, we were not able to detect any ictal discharges during the long‐term scalp EEG. Additionally, the interictal discharges observed were noted in the left frontal area, and most of the brain contusions were observed on the left side of the brain, which is inconsistent with left R/G movement. It was difficult to explain this inconsistency using only the data from the examinations performed. Although we considered performing an intracranial EEG to detect the epileptogenic zone, the patient and his family did not consent to any invasive examinations or treatments other than VNS. Consequently, we were not able to obtain EEG findings that could firmly support the epileptic zone responsible for the R/G movement. Nonetheless, a high‐intensity area was present in the right pre‐SMA, and damage to the right pre‐SMA could lead to the observed symptom. Therefore, based on these findings, and the previous ES study reporting R/G movement,^3^, ^4^ we concluded that the right pre‐SMA may contain the epileptogenic zone in this patient.

## 4 CONCLUSIONS

We detected R/G movement, which has previously only been reported as the consequence of ES, as part of a spontaneous epileptic seizure in a patient who had suffered traumatic brain injury. The epileptogenic zone in the patient was possibly related to the right pre‐SMA.

## CONFLICT OF INTEREST

The authors have no conflict of interests to report.

## AUTHORS CONTRIBUTION

HT: wrote the manuscript with support from SK, KT, TY, YY, AH, FK, and KI. SK and HT: conceived the original idea. SK, KT, TY, YY, AH, FK, and KI: supervised the project.
